# Health system reforms and the needs of the ageing population—an analysis of recent policy paths and reform trends in Finland and Sweden

**DOI:** 10.1007/s10433-022-00699-x

**Published:** 2022-04-15

**Authors:** Liina-Kaisa Tynkkynen, Jutta Pulkki, Leena Tervonen-Gonçalves, Pär Schön, Bo Burström, Ilmo Keskimäki

**Affiliations:** 1 Faculty of Social Sciences, Health Sciences, Tampere University, Tampere, Finland; 2 Department of Social Sciences and Philosophy, University of Jyväskylä, Jyväskylä, Finland; 3 Aging Research Center, Karolinska Institutet and Stockholm University, Stockholm, Sweden; 4 Department of Global Public Health, Equity and Health Policy Research Group, Karolinska Institutet, Stockholm, Sweden; 5 Finnish Institute for Health and Welfare, Helsinki, Finland

**Keywords:** Older people, Multiple care needs, Equity, Health care reforms, Primary care, Health policy, Finland, Sweden

## Abstract

Population ageing with an increasing number of people experiencing complex health and social care needs challenges health systems. We explore whether and *how health system reforms and policy measures adopted during the past two decades in Finland and Sweden reflect and address the needs of the older people*. We discuss health system characteristics that are important to meet the care needs of older people and analyse how health policy agendas have highlighted these aspects in Finland and Sweden. The analysis is based on “most similar cases”. The two countries have rather similar health systems and are facing similar challenges. However, the policy paths to address these challenges are different. The Swedish health system is better resourced, and the affordability of care better ensured, but choice and market-oriented competition reforms do not address the needs of the people with complex health and social care needs, rather it has led to increased fragmentation. In Finland, the level of public funding is lower which may have negative impacts on people who need multiple services. However, in terms of integration and care coordination, Finland seems to follow a path which may pave the way for improved coordination of care for people with multiple care needs. Intensified monitoring and analysis of patterns of health care utilization among older people are warranted in both countries to ensure that care is provided equitably.

## Introduction

Universal health coverage means that all people have access to the health services they need, when and where they need them, without financial hardship. Access to health care also links to the third goal of United Nations Sustainable Development Goals which is to *“ensure healthy lives and promoting well-being for all at all ages” (*UN 2021*)*. At the same time, health inequalities and unequal access to care are challenges faced by most of the health systems around the world (GBD 2018), including the Nordic countries (Keskimäki et al. 2019; Burström 2009; Mackenbach 2020). To reduce inequalities between and within different societal groups, health care policies need to be sensitive to heterogenous needs of different population groups.

There is currently no empirical consensus on the impact of increasing longevity on the need for health care (Greer et al. 2021). According to medicalization theory, longevity increases the number of people in need of care. Due to the population ageing and epidemiological transition, the number of older people experiencing chronic conditions with co-morbidities is increasing. Older people increasingly survive their diseases, but often with chronic health problems and increased care needs (Parker and Thorslund 2007). Another theoretical approach, compression theory, assumes that the population lives longer in good health due to healthier lifestyles and living environments, and service needs accumulate for a shorter period to the end of life. In most cases, people with multiple care needs and functional and cognitive difficulties are aged 80 and older. This age group is growing most rapidly in many countries (StatFin 2021; Statistics Sweden 2018). It is, however, unclear how well the content and suggested measures of health system reforms reflect the needs of older people. It has been argued that health policies are designed for the “younger old” but applied to the “oldest old”, whose care needs are the greatest (Gilleard and Higgs 1998).

In this article, we explore whether and *how health system reforms and policy measures adopted during the past two decades in Finland and Sweden reflect and address the needs of the older people.* The analysis builds on “the most similar cases” drawing on the idea that the Nordic countries represent national contexts that rely on the principle of universalism, public funding, (mainly) public provision and services of high quality. Finland and Sweden form an interesting pair to compare as their population is ageing faster than that in other Nordic countries. Finland and Sweden have also been shown to be similar when considering the supply side resources, public–private mix, access regulation, primary care orientation and health system performance management (Reibling et al. 2019) or the degree of decentralization of health care services (Larsen et al. 2020). Taking this as a starting point, we reflect health care policy developments against the characteristics of health systems we have identified from the scholarly literature to be important in addressing the needs of the older people.

## Older people’s care needs and related care system characteristics

To meet the needs of older people independent of their socioeconomic status, *affordability* of health care is of major importance. Socio-economic differences in health are evident at older age: individuals with higher education usually have better level of health and functioning than those with basic education (Enroth et al. 2019). In addition, people in the lowest income group report more often to have a need to visit a medical doctor compared to those in the highest income group (Hannikainen 2018). However, the ability to visit a doctor may depend on the income level as people in the lower socioeconomic groups may refrain visiting a doctor because of financial reasons (Hannikainen 2018; Molarius et al. 2014). For older people, user fees and other out of pocket payments (OOP), including payments for treatments and medication, may cause problems in terms of affordability and thus accessibility of health care. The research indicates that financial hardship is more likely to occur when public spending on health is low relative to gross domestic product (GDP) and OOPs account for a relatively high share of total spending on health (Tervola et al. 2021). This suggests the need to strengthen the mechanisms for financial protection for older people with chronic diseases and multiple care needs.

Both multimorbidity and decreased functional ability increase the need of different types of care and require joint efforts from a range of care providers (Hujala et al. 2017). Dementia and other memory disorders form great challenges for health and care systems worldwide (Banerjee 2013). In Finland and Sweden, dementia is the dominating cause of admission to residential long term (Sköldunger et al. 2019; Kehusmaa et al. 2018). However, most people with memory disorders live and are cared for at home. A key health system characteristic in this respect is *care integration and coordination* across the care continuum. Coordinated and integrated interventions have shown to be important especially for people with multimorbidity and multiple care needs (Eklund 2009). For many older people, coordination of care is important also because of the cognitive impairments that make them incapable to navigate in often complex service systems. Memory disorders increase with age (Jylhä et al. 2019) and the absolute number of people with dementia has increased due to longevity (ibid.; Banerjee 2013). Dementia and other memory disorders form great challenges for health and care systems worldwide (Banerjee 2013). For example, both in Finland and Sweden, dementia is the dominating cause of admission to residential long term (Sköldunger et al. 2019; Kehusmaa et al. 2018). However, most people with memory disorders live and are cared for at home.

One of the keys for successful coordination and continuity of care is the existence of a *strong primary care system* which can take responsibility of coordination of care. Strengthening and improving primary care systems have become a key strategy to respond to changing population needs (Kuhlmann et al. 2017). Composition of organization of primary care varies country by country but regardless of how the services are organized primary care often is the first point of contact in a health system. Increasing number of older people underscores the need to provide services that can deal with the needs of the population, making the primary care a focal point for health policy. (Groenewegen et al. 2015.) Primary care is generalist care, which focuses on the person as an integral whole (Kringos et al. 2015). This is crucial especially for older people with multiple chronic diseases which also make polypharmacy (the use of five or more drugs concurrently) common among older people (Midao et al. 2018; Johnell and Fastbom 2012). The prevalence of polypharmacy has also increased over time (Gransjön-Craftman et al. 2016; Pulkki et al. 2019). Older people’s extensive use of drugs and high prevalence of comorbidities substantially increase the risk of adverse drug reactions, hospitalizations, and mortality (Johnell and Fastbom 2012). A well-functioning primary care may alleviate these risks.

## Methods and materials

Our empirical analysis draws on a review of the scientific literature as well as “grey” literature, policy documents and descriptive statistics. The timeline for the analysis is 2000–2020.

Comparative method has signified different things at different times (Allardt 2004). In a given situation where societies are ageing across the developed world at an unforeseeable rate, it is of relevance to compare how societies have responded to economic, social and human challenges related to ageing. By scrutinizing policy initiatives, strategies and recommendations as well as “failed” reform proposals, we can make incremental changes that are often left outside the traditional, more structural or statistically oriented analysis visible.

By comparing two societies and their health care policy responses to ageing our research represents individualizing comparisons (Tilly 1984). Tilly writes how this type of comparison contrasts a small number of cases to grasp the peculiarities of each case (ibid., 82). This strand of comparative research involves discovering how different two cases are. So does our study. In the context of comparing culturally, economically and politically similar societies and their responses to ageing, it makes sense to focus on differences rather than similarities.

In terms of empirical analysis, our research consists of three parts. First, we identified the core characteristics of good care for the elderly from the previous research literature (see above). These were 1) health care system capacity, resourcing, and affordability of health care, 2) integrated care and care coordination, and 3) strengthening primary care.

Second, we analysed health care system capacity, resourcing, and affordability of health care by reviewing the key health system indicators and the research literature. The analysis thus started by mapping the context and system characteristics by providing descriptive statistics obtained from OECD Country Health Profiles 2019 for Finland and Sweden (OECD 2019a, b), OECD Health at Glance publications (OECD 2020, 2021), Statistics Sweden, Statistics Finland and Nordic Welfare Database.

Third, we focused on integrated care and strengthening primary care and searched how these aspects are manifested in the selected policy documents. In the search and selection of the key policies, we used Health Systems in Transition repots for Sweden and Finland (Anell et al. 2012; Keskimäki et al. 2019) and Immergut et al. (2021) which provides the description of the major health reforms also in Sweden and Finland. In addition, we used purposive sampling to include a few other initiatives that were not included in the aforementioned publications but which the authors knew to be relevant especially from the point of view of older people or which were so recent that they were not included in the publications. By analysing documents, our research mainly focuses on analysis of policy agendas. The rational-linear conceptualizations of policy-making process see agenda-setting only as a first stage of the process, which is then followed by implementation and evaluation. We follow a constructivist perception of policy-making and argue that while terms and concepts appear on agendas, they play an essential role in constituting the reality by determining how the issue gets defined, framed, and understood. (Tervonen-Gonçalves 2013.) The analysed documents were divided into (1) legislative documents, (2) national level programs, and (3) national guidelines and recommendations (compare for instance Wadman et al. 2009). Also, reform proposals (4) are included if they have had a major impact in health policy developments in a country. The laws, programs and documents are listed in Tables 3 and 4. Because, the needs of older people are often complex, and they require services from various sectors we refer, when relevant, also to services, such as home care and residential long-term care, that are organized under social services in Finland and Sweden but are of special attention of another paper in this special issue (Rostgaard et al. 2022).

### Describing the context

The Finnish healthcare system is built on three partially parallel systems (Keskimäki et al. 2019): the core system is formed by a tax-funded system run by municipalities and hospital districts. In addition, there is an obligatory social and health insurance system reimbursing, for instance, the use of private health care and prescription medicine, and an occupational health care system for employed people. The core system is financed through municipal taxation, state transfers and user fees. Municipalities (*n* = 297, mainland Finland) are responsible for both health care and social services (including home care and long-term care) and they can organize the services by themselves, together with other municipalities or by purchasing services from other municipalities or from private providers. Specialized medical care is also financed by municipalities, but it is organized through 20 hospital districts which are federations of municipalities. (Keskimäki et al. 2019.)

In Sweden, the system is divided into local and regional levels, health care being the responsibility of the 21 regions and social care the responsibility of 290 municipalities. Both health and social care are tax funded, with regions and municipalities collecting taxes which are complemented by state grants and user fees. The service provision is mainly public but especially in primary care and in larger cities, the number of private providers has increased in recent years (Ekonimifakta 2021; Burström 2017; Svallfors and Tyllström 2018; Anell et al. 2012).

## Health care system capacity, resourcing, and affordability of health care

The main differences and similarities concerning the capacity and resourcing of the health and care systems are provided in Tables 1 and 2. The level of public health care financing is lower in Finland when measured as proportion of GDP or in per capita health care costs. Also, the share of public funding is lower in Finland. The differences are large also in terms of long-term care costs, with per capita costs in Sweden being twice as high as in Finland although the proportion aged 80 + years is the same in both countries.

**Table 1:** Finland has more doctors working in primary care than Sweden, both as a proportion of all doctors and per 100,000 population (*Source*: Larsen, Clausen, Höjgaard. VIVE report 2020)

	Number of general practitioners (GPs)	GPs per 100,000 population	GPs as proportion (%) of all doctors
Finland (2016)	3,950	72.1	19
Sweden (2017)	6,028	58.4	14.9

**Table 2:** Key health system indicators for Finland and Sweden.

	Finland	Sweden
Proportion aged 65 + years (%)	20.2	19.7
Proportion aged 80 + years (%)	5.1	5.1
*Percent in institutions, service housing, or with home care services*		
In institutions 80 + years (%)	14.2	14.1
In institutions 65 + years (%)	5.1	4.7
Home care 80 + years (%)	16.4	24.0
Home care 75–79 years (%)	5.0	7.0
Practicing doctors per 1 000 population	3.2	4.3
Per cent (%) of GDP to health	9.2	11.0
Health expenditure from public sources as a share (%) of total	80	85
Health expenditure from public sources as a share (%) of total government expenditure	14	19
Health care cost per capita (euro)	3036	3872
Outpatient care	1117 (37%)	1303 (34%)
Inpatient care	751 (25%)	848 (22%)
Long-term care	578 (19%)	1024 (27%)
Pharmaceuticals and devices	443 (15%)	478 (12%)
*Prevention*	117 (4%)	126 (3%)
User fees (caps, euro)		
Visits	683	109
Pharmaceuticals	572	218
Transport	300	N/A
Share of households with catastrophic health spending	3.8	1.8
Population reporting unmet needs for medical care (%)	4.7	1.4

The numbers describe mostly year 2019 or nearest available year. Monetary unit for health care expenditure and user fees is Euro. (*Sources*: OECD 2019a, OECD 2019b, 2020, OECD 2021, Nomesco Report 2017)

Out-of-pocket expenditures (OOPs) for social and health care services and medication are relatively high in Finland compared to Sweden when measured in terms of annual payment caps (Table 2). This is an important feature which may result in inequalities in access and catastrophic costs especially among older people in lower socio-economic groups (Tervola and Heino 2020; Ilmarinen et al. 2020). In Finland, larger share of people reports catastrophic health spending compared to Sweden (Table 2). One of the tools for cost containment in Finland has been to decrease the public reimbursement levels and to increase user fees in services despite the relatively high proportion of OOPs already. Several reforms have aimed to curb public spending on medicines (Tervola et al. 2021) which have also included reforms decreasing the reimbursement levels for outpatient prescribed medicines. To adjust user fees, the new Act on social and health care client fees entered into force in July 2021. It extended OOP-free services and it also contains long-awaited stipulations for payments of service housing. (Valtioneuvosto 2020.)

Higher share of people reports unmet care needs in Finland compared to Sweden (Table 2) but in both countries, inequalities in access to care persist. Among persons 65 years and older in Finland, 10% in the lowest income group did not visit a doctor in 2013–2015 because of financial reasons, compared to 3% in the highest income group. (Hannikainen 2018.) In Sweden, only 2% of persons aged 65–84 years refrain from seeking health care for economic reasons (Molarius et al. 2014). In Finland, there are differences in access to health care between low-income and high-income groups: low-income groups use public services with often long waiting times, while high-income groups use well-available occupational health services or private services (Keskimäki et al. 2019). Even though a similar occupational health care services do not exist in Sweden, similar pattern can be discerned: socio-economic differences in morbidity and health care needs are not reflected in corresponding demand and use of health care services (Burström 2009). However, the use of emergency department care among older persons is higher in low-income groups (Doheny et al. 2019), largely explained by their greater needs.

The core problems in health system capacity in Finland and in Sweden have been related to uneven distribution of resources between different health system functions and sectors. Especially primary health care and elderly care have been under resourced. In Finland, in comparison with specialized health services, there is increasing imbalance in funding. In 2009–2019, there was a 30% increase in municipal specialized health care spending, while no change in primary health care funding (THL 2021). Together with staffing problems, this has resulted in unmet care needs (i.e. long waiting times) especially among older people (OECD 2019a). In Sweden, in turn, the funding is allocated with a stronger emphasis for instance in long-term care (OECD 2019b). Despite intentions at national level to increase the capacity in primary health care, the proportion of doctors in primary health care is still less than 15% in Sweden (Larsen et al. 2020) with higher proportion of primary care doctors in Finland than in Sweden (Table 1). However, in general, Finland has less doctors per capita than Sweden (Table 2).

## Mapping the health system reforms and policy measures in Finland and Sweden

In the following, we describe and compare the policy measures that have been taken in Finland and Sweden in the areas of 1) integrated care and care coordination, and 2) strengthening primary care. Tables 3 and 4 are provided to sum up the reforms in each area in 2000–2020.

**Table 3:** Health system reforms in the area of integrated care and care coordination in Sweden and Finland in 2000–2020

Integrated care and care coordination				
	Legislation	Program	Recommendation	Reform proposal
Sweden	Prescribed drug register (2005) Amendment in the Health and Medical Services Act and the Social Services Act, stating that people who need help from both health and social care should be offered a joint individual care plan (2010) Regulated right to annual drug review for persons 75 + , prescribed five or more drugs (2012) Act on Coordinated Discharge from Hospital Care (*Lag om samverkan vid utskrivning från sluten hälso-och sjukvård)* (2018)	Governmental programme on care coordination for older people with complex health problems (2010–2015)		
Finland	Health Care Act (2010) Act supporting the capacity of older population and on social and social care services for older persons (*Vanhuspalvelulaki)* (2013) *Kanta*-Services (The National Patient Data Repository) (2016)	Supporting integrated service concepts and care coordination through national programs (*Kaste-ohjelma 2010–2015*) National strategic programs on aging (1998, 2001, 2020) Future Health and Social Services Centres Programme 2020–2022	National Quality Recommendations on developing services for older people (2001, 2008, 2013, 2017, 2020)	Administrative integration in national reform attempts (SOTE) and local/regional reforms (2007–2018)

**Table 4:** Health system reforms in the area of strengthening primary care in Sweden and Finland in 2000–2020

Strenghtening primary care				
	Legislation	Program	Recommendation	Reform proposal
Sweden	Specification of Waiting time guarantee (2005) Law on choice in health and social care (LOV) (2009) National Choice Reform in Primary Care *(Vårdvalssystem i primärvården) (2010)* (New) Patient Act (2015)			
Finland	Care guarantee (2009) Health Care Act (`) Act Supporting the Functional Capacity of the Older Population and on Social and Health care Services for Older Persons (2013)	National development programs in social and health care (Kaste 2008–2015)	Med75 + database (2015) National Quality Recommendations on developing services for older people (2013, 2017, 2020)	

### Integrated care and care coordination

In Finland and Sweden, integrated care and care coordination are high on the agenda of national policy. However, these have been advanced partly with differing tools and targets. In Finland, restructuring the system through administrative integration, as well as developing services and practices, has been promoted through national programs and legislative initiatives. In Sweden, in turn, the integrated care practices have mainly been promoted through national level programs. Concurrently, choice-and market-oriented competition reforms have been introduced which have at least partly undermined care integration and increased system fragmentations.

In Finland, administrative integration has been at the core of national reform attempts on social and health care. While primary health care and social services are organized in municipalities, the long-lasting aim has been to administratively integrate health and social services at same organizational level and under the same budget (Tynkkynen et al. 2021). The national administrative reform will be implemented 2023 onwards, but already before that, smaller reforms both at national and local levels have been implemented to support the development of integrated care practices (Keskimäki et al. 2018; Tynkkynen et al. 2019). In addition, the Health Care Act (2010) aimed at strengthening the integration between primary health care and specialized health care. Although a legislative basis for integration was created in this law, in practice, its influence as a steering device has been rather weak due to the lack of financial or structural elements included in the law.

Integration of care at clinical and professional level and specifically for older population has been promoted through special legislation or through amending laws in both countries. In Finland, the Act Supporting the Functional Capacity of the Older Population and on Social and Health care Services for Older Persons (2013) aimed to improve older persons rights to access to social and health care services in accordance with their needs. The Act entails an obligation for social and health care authorities to make individual care plans in co-operation with the clients and with their family members. The Act also stipulates that health care professionals should inform social care professionals when discharging an older person from a hospital. In addition, five national recommendations for the quality of care for older people have been issued to support municipalities (STM & Kuntaliitto 2001; 2008; 2013; 2017; 2020) and two strategic programs on aging have been published (STM 2004, 2020a).

In Sweden, similar developments have taken place through amendments in the Health and Medical Services Act and the Social Services Act in 2010. The amendments focused on people who need help from both health and social care and stated that they should be offered a joint individual care plan to ensure the service continuity and patient safety (Samordnad Individuell Plan, SIP). The Act on Coordinated Discharge (2018) from hospital care also stipulated that patients who need social and health care after discharge from hospital should be provided with an individual care plan.

In addition to legislative tools, the development of integrated practices in both countries has happened through *national programs* which have also included financial support through state grants. In Finland, the National Development Programme for Social Welfare and Health Care KASTE 2008-2015 (STM 2008; 2012) strived to promote integration through intersectoral cooperation and integration by supporting the development of individual care and service plans, chronic care models, and descriptions of service chains. These practices have been adopted in many municipalities and it can be said that most of the developments in terms of integration have taken place through bottom-up developments of integrated practices (Sinervo et al. 2016). Future Health and Social Services Centres Programme, launched in 2020, works towards similar goals by developing integrated practices at primary care level by funding regional development work and providing national support (STM 2020b).

Similar developments have taken place in Sweden where several national programs have been introduced to improve integration of services and care coordination. In 2010–2015, the government initiated a programme to improve care coordination for older people with complex health problems by introducing financial incentives to enhance the use of quality registers and to reduce hospital admissions, readmissions and to reduce inappropriate drug use among older people. This programme aimed at improving coordination between regional health care services and municipal social services for older people (Hagman et al. 2014). The initiative was resourced through state grants to regions and municipalities to develop the services in five areas: a preventive way of working; good care of dementia; good care at the end of life; good pharmaceutical treatment of older people; and coordinated health and social care.

In Finland, risks related to polypharmacy and the use of inappropriate medicines have been aimed to reduce through free-accessed Meds75 + database of medication for older persons. The purpose of the database is to support the clinical decision-making on the pharmacotherapy of patients over 75 years of age and to improve medication safety especially in primary health care. (Fimea 2021.) The Meds75 + database has been maintained by the Finnish Medicines Agency, Fimea since year 2015 (Fimea 2016). Also, major development in terms of supporting integration of information systems has been implemented in the form of the National Patient Data Repository called *Kanta Services,* which is an electronic patient record covering entire population. It includes all public and private health care providers, enables electronic prescription of medicines, and provides clinical information not only to medical professionals but also to patients. (Larsen et al. 2020, 80).

During the 2000s, several prevention policies regarding polypharmacy were developed and implemented also in Sweden. One example was an intervention to improve drug therapy for older people. In 2005, the Swedish prescribed drug register was initiated, which include data on all prescription drugs dispensed to the Swedish population. One aim with the registry was to evaluate the quality in drug treatment among older people (Johansson and Schön 2017). Evaluations have shown significant improvements, for example an almost 40% reduction in drug use between 2005 and 2013. These results have led to regulations on drug reviews. People 65 years and older who are prescribed five or more drugs have the right to a drug review (Fastbom and Johnell 2015).

### Strengthening primary care

Improving access to primary care services has been high on the agenda during the analysed period in Finland and Sweden. However, the countries have adopted differing paths. In both countries, care guarantee legislation has been used to improve access to primary care. In Finland, maximum waiting times for primary and specialized health care were introduced through national care guarantee that initially entered into force in 2005. If health centres cannot provide the service in due time, they must obtain the service from other service providers, such as private providers without any additional cost to the patient. However, in Finland, the waiting times set by care guarantee are relatively long with maximum waiting time to non-urgent care at primary care being three months (Health Care Act 2010). In Sweden, waiting-time guarantee was introduced already in 1992, and it was further specified in 2005. The legislation was further promoted through the “Queue-billion (*Kömiljarden*)” initiative starting from 2008. The aim of the initiative was to provide financial incentives to the regions to meet the targets set in the care guarantee. These incentives were discontinued in 2014 but in 2019 revived again by the national government.

To strengthen primary health care, Sweden has also introduced other legislation to increase the supply of primary health care services, through privatization and market-oriented reforms, which have been introduced over a longer time, by different mechanisms and small changes in legislation (Svallfors and Tyllström 2018; Dahlgren 2018; Wingborg 2017). The law on choice in health and social care (LOV) came into force in 2009 with a purpose to enable establishment of new private providers and facilitate choice in social and care services. Applying the law is voluntary for municipalities, but the law paved the way for the subsequent mandatory law on Choice in Primary Care. In 2010, an amendment was made to the Health and Medical Services Act, mandating the regions and county councils to allow citizens to choose their PHC provider, and to allow private providers to freely establish practices if they met certain pre-defined criteria. All regions had to change their system to provide choice in primary care. In effect, both pieces of legislation mean a voucher system, where residents having the right to the service can choose the provider they want, and the municipality (for social service) and the region (for primary care) are responsible for accrediting the private providers and for paying for them.

In 2019, a government bill, “Close care” (Nära vård), proposed that primary care must be strengthened to be the focal point of care, and the link to other specialist care (Swedish government 2019). However, no major changes have yet occurred. In a follow-up, the National Board of Health and Welfare concluded that the COVID-19 pandemic had been a hindrance to implementing the reform, along with difficulties to recruit staff, especially to rural areas. However, some regions and municipalities were reported to have increased their collaboration and there was an increased use of e-health services. Indicators to follow up the implementation of the reform and their impact will be developed (National Board of Health and Welfare 2021).

To strengthen the supply of primary care services, the “Swedish type” choice and competition model was also proposed in Finland in 2016–2018 (Tynkkynen 2021) but Finland has adopted a slightly different pathway to strengthen the services at primary level. The Health Care Act (2010) is the main piece of legislation regulating both primary health care, specialized health care and promotion of health and well-being. When enacted, the explicit aim was to strengthen primary health care. Finland has a wide network of municipal level primary care units which provide primary care services. In many places also social services are operating in the same centre. Integrating larger set of services under the same unit has indeed been one of the local strategies to strengthen primary health care in general (Sinervo et al. 2016) and these initiatives have been supported by several national programmes, especially Kaste Programme I (2008–2011) and Kaste Programme II (2012–2015), which especially focused on reforming the structure and content of services targeted to older people. In practice, this mainly meant promoting different solutions that supported living at home. The most recent national programme to strengthen primary care is *The Future Health Care and Social Services Centres*-programme (STM 2020b). The programme aims at shifting the focus of services from specialized health care to primary health care and to prevention.

Apart from being part of the social and health care reform agendas in Finland, primary health care has been implicitly on the agenda of old-age care policy too. All recommendations for the quality of care for the older people (2001, 2008, 2013, 2017, 2020) and above-mentioned strategic programs on ageing (2004, 2020) as well as Act Supporting the Functional Capacity of the Older Population and on Social and Health care Services for Older Persons (2013) seek to increase the proportion of older people living at home with adequate home care and to reduce the number of those aged over 75 years living at residential long-term care. Implicitly, this applies also to primary health care and home health care as part of it. Despite the desire to shift the balance of care from institution to home care, the proportion of people receiving home care or family support is still below the national target. It has also been argued that service structures for regular home care have not achieved the quality recommendations that are outlined in the Act Supporting the Functional Capacity of the Older Population and on Social and Health care Services for Older Persons. (Rissanen 2020, 37).

## Discussion

Our analysis shows that there are similarities but also several differences in terms of resourcing and capacity of health systems and in the policies made in areas of primary health care and care integration in the two countries. Similarities relate especially to national level steering through programs that aim at supporting service development at local level and which often include financial incentives. Also, national care guarantees have been used to improve access to primary care, but which have not been alone sufficient to improve the access to care. Both Finland and Sweden have a high degree of decentralization and relatively weak national governance. This may explain why certain initiatives, even regulations, remain intentions and do not materialize as intended if financial incentives are not included in the reform.

The differences between the countries relate both to the elements of the current system (capacities and affordability) and to the general policy developments that have taken place. When it comes to current systems, we can observe that Sweden invests in health and care systems more than Finland does, and the level of OOPs is also higher in Finland than in Sweden. In Finland, people also report more unmet care needs and catastrophic health spending compared to Sweden. What is also remarkable is that in Finland, cost containment has been high on the national policy agenda despite the already lower level of spending and higher OOPs (Tynkkynen et al. 2021). In terms of cost containment, both countries have emphasized outpatient care and community living (“ageing in place”) but in Sweden, the resourcing in long-term care remains higher than that in Finland.

One of the reasons for under resourced primary health care in Finland may stem from the existence of dual practice for doctors and occupational health care system organized by employers which provides extensive ambulatory services for a large proportion of the working age population (Keskimäki et al. 2019). Retired older people are not covered in the occupational health care system, which makes strengthening public primary health care system of high importance to safeguard adequate services for people at all ages.

In both countries, national level programmes with financial incentives or grants have been used to steer the local and regional level. However, the goal to strengthen integration between different actors and levels of the system also through structural reforms seems to be more evident in Finland. Achieving greater administrative integration in health and social service systems has been a goal in various reform proposals and policy initiatives in the last two decades in Finland. The separate organization of primary and specialized care and social services, particularly in the context of an aging population, is seen as an obstacle to improving health system performance. Several governments have attempted fundamental systemic reforms with three core objectives: (1) centralization of organizational structure, (2) improving access to primary care, and (3) integration of services. (Keskimäki et al. 2019; Tynkkynen et al. 2021.) The persistent attempts to reform the social and health care systems during the last two decades have kept the aforementioned issues high on the political agenda and there have been local and regional initiatives to promote these goals (Keskimäki et al. 2018). In the summer 2021, the parliament of Finland enacted new legislation that follows the policy path that has already been visible both at national and local initiatives. From 2023, the Finnish health system structures will be reformed transferring responsibility of all health care and social services to 21 independent counties which will receive funding from the state budget. Therefore, at least in the case of Finland, to analyse how the identified key components of good care have been considered in health care reforms, we must not only look at those major reforms that have been implemented, but also consider the incremental steps and fuzzy policy paths taken along the preparation of failed major reforms.

In Sweden, the structural reforms have not been high on the policy agenda. The responsibilities and funding of services are divided between regions (health care) and municipalities (social services). Especially for persons with a need of services from both sectors, this may pose challenges in the form of care coordination and limit the possibilities for the state to govern the overall system. From the point of view of older people, the structural problems in the system were materialized in the wake of COVID-19 pandemic. Poor coordination between different sectors and levels of the system may be problem also when a system faces a sudden shock (Sagan et al. 2021). A recent investigation by the Swedish Corona Commission was very critical of the lack of coordination between national authorities and the regions and municipalities and the fragmented system for long-term care of older people (Coronakommissionen 2020, Schön and Fritzell 2021). Also, in Finland, local and regional authorities have reported problems in collaboration across sectors and levels of the system which has also emphasized the benefits of structural and organizational integration especially from the point of view of people with complex needs. COVID-19 pandemic has also emphasized that while national steering is, in many respects, focal for improving the overall performance of the system, local action matters and thus, in the future studies, it would be important to focus also on local and regional agendas and development initiatives. (Kihlström et al. 2021.)

Another dimension to structural differences between the countries concerns different developments in the supply side of primary care. In Sweden, the choice and competition reform (Vårdval) was introduced in 2010 which has further fragmented the structure of the primary level services. While the reform has increased the supply of primary care services in some regions (Isaksson et al. 2016), the legislation has not improved, but rather complicated, the coordination of the services for the people with multiple care needs (SOU 2016; Burström et al. 2017). The choice reform in social services also meant that older persons who are granted home help, and who have a private provider, can top up and purchase additional services by that provider to half of the cost via tax rebate. Public providers are not allowed to offer topping up services. This has been questioned as it may lead to differentiated quality and cause inequalities in care (Szebehely and Meagher 2018).

In this paper, we have provided description of health system capacity and affordability as well as description of policy developments in the areas of primary care and integrated care over the past two decades in Sweden and Finland. Given the large and rapid increase in people aged 80 years and above in the coming decade in both Finland and Sweden, it will be important to monitor and analsze patterns of health care utilization among older people in relation to these and future policy changes. In future studies, it would also be important to analyse the target population the national and local decision-makers have in mind (Pulkki and Tynkkynen 2016). It has been argued that health policies can be designed for “younger old” but applied to the “oldest old”, whose care needs are the greatest (Gilleard and Higgs 1998). To ensure that care is provided equitably in relation to need and that those with the greatest needs are given the priority also health and care systems need to be seen as an investment. COVID-19 has shown how well-functioning welfare systems are the best insurance the societies can have when facing a public health crisis (Sagan et al. 2021). This lesson should be cherished also in “normal” times to tackle the persistent inequalities (over time and over life-course) that exist in health system also in the Nordic countries which are often praised for their universalism and solidarity. It is important to understand that tackling inequalities in ageing means tackling them already in younger ages (Greer et al. 2021). Thus, while strengthening health system capacity, affordability, primary care and care integration is of importance for older people’s services, they potentially work towards improving health system performance over the life course and thus contribute towards tackling inequalities in the society as a whole.

## Conclusions

In this paper, we have investigated health system affordability, resourcing and capacity as well as the national policies that have been adopted to strengthen primary care, care integration and coordination in Finland and Sweden. The countries share similar goals, but the capacity to answer to growing needs of older population and the measures taken have been different and sometimes even undermining the actual policy aims especially from the point of view of older people with multiple care needs. In Sweden, the health system is better resourced, in general, and the affordability of care is better ensured through relatively low levels of OOPs. However, the choice reforms in primary care and social care in Sweden do not primarily address the increasing group of older persons with complex needs, who would rather benefit from integrated services. In Finland, OOPs are relatively high and the level of public funding is lower which may have negative impacts on people who need multiple services. However, in terms of integration and care coordination, Finland seems to be in a better place to improve the system also through structural reforms which may pave the way for improved coordination especially for people with multiple care needs. Intensified monitoring and analysis of patterns of health care utilization are needed to ensure that care is provided in an equitable way.
